# How beliefs in traditional healers impact on the use of allopathic medicine: In the case of indigenous snakebite in Eswatini

**DOI:** 10.1371/journal.pntd.0009731

**Published:** 2021-09-09

**Authors:** Sarah Nann

**Affiliations:** Manchester Metropolitan University, Manchester, United Kingdom; College of Health Sciences, Bayero University Kano, NIGERIA

## Abstract

Snakebite is a major public health problem in Eswatini and serious envenomations can be responsible for considerable morbidity and mortality if not treated correctly. Antivenom should be administered in hospital in case of adverse reactions and any delays due to distance, transport, costs, antivenom availability and cultural beliefs can be critical. Myths and superstition surround snakes, with illness from snakebite considered a supernatural phenomenon best treated by traditional medicine since healers can explore causes through communication with the ancestors. Traditional consultations can cause significant delays and the remedies may cause further complications. Four rural focus group discussions were held in varying geographical regions to establish why people may choose traditional medicine following snakebite. The study revealed four themes, with no apparent gender bias. These were ‘beliefs and traditions’, ‘logistical issues’, ‘lack of knowledge’ and ‘parallel systems’. All snakes are feared, regardless of geographical variations in species distribution. Deep-seated cultural beliefs were the most important reason for choosing traditional medicine, the success of which is largely attributed to the ‘placebo effect’ and positive expectations. Collaboration and integration of the allopathic and traditional systems assisted by the regulation of healers and their methods could improve future treatment success. The plight of victims could be further improved with more education, lower costs and improved allopathic facilities.

## Author summary

There is a high incidence of snakebite in the rural areas of Eswatini and victims often seek traditional remedies or visit traditional healers as the first treatment step. This can cause a delay in the administration of antivenom needed for serious bites, leading to traditional healers often being held responsible for tissue damage, amputation or even death. Interviews with different rural community groups were conducted to investigate why people would initially choose the traditional rather than the conventional treatment route to treat snakebite. No gender bias was found and results indicated that powerful beliefs are the main reason, enhanced by myths and superstitions which suggest snakes are of supernatural origin. They are also regularly used in witchcraft and any snake related illness is believed to require supernatural healing. Such strong beliefs are consistent throughout Eswatini illustrated by the black mamba being considered the deadliest species even in areas where it is rarely seen. Logistical issues such as transport, cost and inadequate healthcare facilities together with poor education further contribute to the choice of a traditional treatment route. Snakebite management could be aided by improvements in these areas and as healers are both respected and accessible, collaboration and integration between the two treatment systems would further improve the outcome of victims.

## Introduction

### Background

Snakebite is a major public health problem in the rural communities of Eswatini and other countries in sub-Saharan Africa [1]. In 2017 it was re-added to the WHO’s list of neglected tropical diseases [2] with annual estimates of envenomings in southern Africa of over 314,000 bites, 7000 deaths and 5,900 amputations [3]. Disabilities from snakebite include disfigurement and blindness, as well as psychological effects such as anxiety and post-traumatic stress disorder. Pregnant women may miscarry, especially if antivenom treatment is absent or delayed. Victims may also suffer high medical costs and a loss of earnings [4,5,6].

Eswatini is a land-locked country measuring approximately 17,000km^2^ with an estimated population of over 1.3 million. The climate ranges from temperate in the western highlands (highveld), to hot and humid in the lowland regions (lowveld) with summer rainfall throughout the country. The economy is primarily agricultural with sugar being the main export [7]. The formal (allopathic) healthcare system is comprised of around 14 hospitals, 5 government health centres and 6 public health units. There are also 215 clinics and outreach sites managed by nurses and located mainly in rural areas. The informal healthcare system is made up of traditional health practitioners or healers [8]. The country is home to 61 species of snake, 7 of which may cause severe envenomation. These include the black mamba, (*Dendroaspis polylepis*), or ‘ímamba*’* in local siSwati, the Mozambican spitting cobra (*Naja mossambica*) or ‘mfeti’, the snouted cobra (*Naja annulifera*) and the rinkhals (*Haemachatus haemachatus*), both known as *‘*phemphetwane’, the puff-adder (*Bitis arietans*), or ‘libululu’, the boomslang (*Dispholidus typus*), or “indlondlo*’* and the vine snake (*Thelotornis capensis*), or ‘lununkhu’ [9]. Snakebites occur more often in warmer, lower regions [10] especially in areas where the natural habitat is developed for agriculture [11]. Many bite incidences happen whilst walking, working in the fields and hunting, as well as sleeping and using outside toilets [12,13]. In Eswatini’s low-lying sugar cane farms the venomous puff-adder, Mozambican spitting cobra and the much feared black mamba are common.

Serious bites require antivenom which must be given at a hospital as there is a high risk of anaphylaxis, occurring in 19–39% of cases in a South African study [10]. Any delay between its administration and the bite occurring can be critical [13] with an hour delay increasing the risk of death by 1% [4]. A community study in Myanmar illustrated reasons for such life-threatening delays, these included the influence of cultural beliefs, transport issues, cost factors, and a poor supply or incorrect use of antivenom [14]. Studies in South Africa [2] and Nigeria [4] found snakebite victims often first consult traditional healers or self-medicate using traditional remedies. This can delay antivenom treatment and lead to further medical complications.

Much folklore and superstitions surrounding snakes are passed down through the generations, many believe snakes represent spiritual ancestors or appear as the result of a bewitching [11,15–17]. Apprehension of snakes may originate from an evolutionary fear response [18–20] and bites or encounters may cause anxiety or post-traumatic stress disorder [6,17]. Traditional healers are believed to mediate between the living and the dead and with their medicines restoring spiritual harmony they can cure illness, counteract witchcraft and protect the living [21–25].

### Traditional healers

Traditional healers are highly respected and influential in both rural and urban southern African communities [26,27]. It is not known how many currently exist in Eswatini but a local study found up to 85% of the population, both educated and non-educated, seek their help [28,29]. In neighbouring South Africa a study found eight times as many healers as allopathic doctors [22], although their use may be declining [30], and in Mozambique the ratio was even greater [22].

In Eswatini and other parts of Africa there are three main categories of traditional healer [31]. The ‘Sangoma’ are mainly women diviners who communicate with ancestral spirits [32,33]. They are ‘called’ to their position by the ancestors and identified through exhibiting an ‘initiation’ illness, often headaches, psychosis or other apparently incurable symptoms [34]. The ‘Inyanga’ are predominantly men who practice the art of ‘kunyanga’ meaning to ‘heal’ or ‘treat’ using herbs and / or animal parts. A ‘Lugedla’ is a senior Inyanga who can initiate the Sangoma and Inyanga, they include the witchdoctors and can be identified by their dreadlocks. The Umprofethi or Umthandazi are spiritual/faith healers and prophets who use prayer, holy water, teas and ash to heal [22]. Treatments by the different types of healers may overlap [21] and communities generally have their preferred remedies. Traditional medicine is known as ‘muti’ and can be ingested, washed in, smeared on or smoked, or may be in the form of enemas or gastric purgatives. Muti used to prevent or treat snake-related illness often contains snake body parts [35] and snake ‘repellents’ can also be carried on the person or sprinkled on the ground [15,36]. Muti may be free of charge, or if successful the price might be a cow [22].

The success of traditional medicine is believed to be largely due to the placebo effect. A placebo is an ‘ineffective’ medication which leads to mental and/or physiological changes in patients because they believe it will be successful. Negative placebos (‘nocebos’), often used in witchcraft, are similarly driven by beliefs but have the opposite effect causing increased anxiety and illness [37]. Reasons behind the placebo effect are still unclear. Various theories have been proposed with supportive neuroimaging studies to explain how psychological activities such as beliefs and emotions interact with neurological processes, affecting physical and mental health [38]. The term ‘placebo’ has been broadened to include the rituals of muti preparation and administration as well as the treatment itself. Beating drums, chanting and sacrifices to summon and appease ancestors create an emotionally charged atmosphere to enhance the medicinal effects [37–39]. Treatment effectiveness is further influenced by the highly symbolic physical characteristics of plants and animal parts [40] and also depends on the type of symptoms, with pain reduction and depression being the most commonly improved. Individual differences in attention, compassion, anxiety, genetics, learning mechanisms and personality traits can also affect the treatment success [39,41] and a successful outcome is maximised by a positive interaction between a confident healer and a believing patient. Usually lengthy consultations include counselling which reduces stress, and may assist in treating psychiatric conditions thought to be caused by a ‘bewitching’. Placebos are known to activate the same nervous, immune and endocrine pathways as pharmacological drugs but since their effects are inert their dosage does not need to be as regulated [27]. Some plants do possess drug-like molecular properties to help reduce inflammation [40] which, combined with the placebo effect, lessens the pain and stress associated with snakebite, however the venom toxins within the body can only be neutralised by administering antivenom. Traditional and allopathic treatment systems are sometimes used together to treat illnesses [31,42].

The success rate of traditional healers for treating snakebite is hard to assess because documentation is scarce, this is partly because their methods are secret and committed to memory [43]. They do not keep records and are not obliged to notify or engage local health departments. Whilst hospital cases are recorded they do not accurately represent the number of snakebite cases or mortality in the region [44]. Studies indicate only half of the patients reach medical facilities [45] and many have received (often unsuccessful) traditional treatments beforehand [13]. Consequently there is little information about victims who only used traditional medicines, used no medicines at all or those who died before arrival at the hospital. A study in Myanmar indicated a 95% success rate for traditional healers [14] but some bites may have been from non-venomous snakes with no envenomation, or may have been ‘dry’ bites with little or no venom injected. Traditional medicine may also be preferred because of distrust in allopathic medicine, where cost and supply chain issues may mean victims die or become disabled through a shortage of antivenom [4,11,46]. This distrust may also arise because of poor allopathic outcomes due to reactions to antivenom, late referrals by healers and late arrivals at hospitals [44,47,48].

### Traditional remedies

Information regarding traditional remedies for snake-related treatment in Eswatini is scarce. Traditional healers are usually the initial treatment pathway [28] with *Strychnos madagacariensis* or ‘umkhwakhwa’ leaves documented as being one of their medicines [43]. Most treatment data come from hospitals in neighbouring South Africa, where two studies also found that 80–90% of snakebite victims initially either visited a healer or self-medicated with traditional medicine. Scarification or ‘xaba’ was performed in over 30% of cases studied, usually with a razor blade or sharpened cow horn around the bite site with herbal mixtures being rubbed in, can result in infection and bleeding complications. ‘Isibiba’ was found to be one of the most common treatment for snakebite and although its effects are unclear, 80% of victims used it prior to arriving at hospital. It is made from a mixture of cremated, ground snake parts and herbs and used as a ‘lick’ or drink or applied topically with other agents including household detergent, potassium permanganate, paraffin, breast milk and snuff [13,49]. An earlier study found scarification rather than isibiba was the most common traditional treatment [50]. This could be due to more focus being on the healer administering the remedy rather than on self-treatment, or the study being conducted in a region where isibiba was less popular [49], or because there was less HIV/AIDS awareness at that time. Other common oral treatments for snakebite include drinking urine, (mainly their own) and eating plants such as crushed aloes to induce vomiting [43,49]. Complications from traditional remedies include poisoning and a condition from enemas that allopathic doctors termed ‘ritual enema induced colitis’ [25,35,51]. Research found tourniquets were the most common traditional treatment (83%) in both South African and Kenyan research [45,49] although these were not highlighted in other later studies [13,14] perhaps since they are considered to be an allopathic treatment, or people may have become more aware of their associated medical complications. Whilst tourniquets may help temporarily with some neurotoxic envenomations they can exacerbate cytotoxic symptoms [52] and must be used with care. Other traditional snakebite treatments include digit amputation and the application of a ‘healing stone’ [53], and breast milk and urine are recommended for venom sprayed into the eyes [49].

Some believe that a reduction in traditional healer consultations could help resolve the snakebite problem [10], achieved by improving the allopathic system such as having more antivenom and trained staff available [11]. Visits to healers might be reduced if people in rural communities were linked to allopathic health centres, rather than a traditional healer being their only obvious treatment option [4]. However people may resist being ‘converted’ because of powerful traditional beliefs and such efforts may instead cause tension and disputes [14,46]. Much needed community research will help determine why snakebite victims do not reach allopathic care timeously [10]. Qualitative research can produce a body of rich data from a small number of participants, in this case through focus groups, which quantitative survey data could not provide [54]. This Eswatini project aimed to improve the plight of snakebite victims by investigating community perspectives on snakes and snakebite together with the two treatment systems.

## Methodology

### Ethics statement

Ethical clearance was obtained from the Manchester Metropolitan University ethics committee (Psych REC Ref. No.:8080). Prior to discussion participants were made aware of the project aims, their protection and the right to withdraw and their informed written consent was obtained. They were debriefed afterwards and provided with contact details of the researcher, the project supervisor and a counsellor should they later have any queries or experience any distress. A translator was available to explain the forms and to interpret where necessary during the discussion. Snakes are known to elicit a potential fear response [18] and to ensure the well-being of participants a counsellor and a church pastor (in consideration of local culture) were present throughout the session in case anyone became distressed. A distress protocol was also put in place. Light refreshments were provided as participants arrived and chocolates were given afterwards to participants and helpers as a token of appreciation. Participants were allocated batons of different colours on arrival, used to identify them during the session thus maintaining anonymity. Their data was kept confidential, stored on a password-protected computer and was further anonymised through generating a unique code recorded on the debrief sheet should anyone wish to leave the study during the withdrawal period.

### Participants

**Fig 1:**
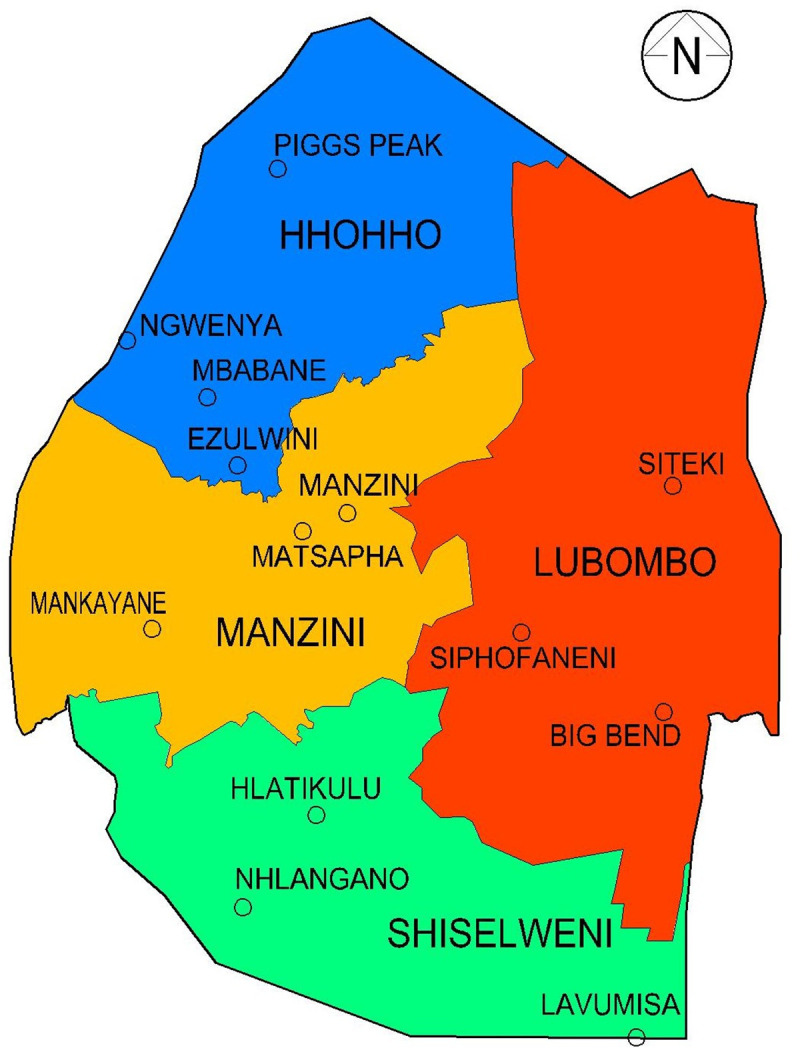
Regions and Towns of Eswatini.

The study was advertised and a total of 32 participants, eight from each of the four rural areas, attended the focus group sessions. Purposive sampling was used to obtain a range of thoughts and experiences. Permission was first sought from the village ‘Elders’ and adverts were prominently displayed notifying villagers of the study with the details of the community counsellor for registration. The inclusion criteria were adults over 18 years of age and inhabitants of the community, whilst the exclusion criteria were children and non-residents of the village. The four communities were Pigg’s Peak (PP), (highveld), Mankayane (M) and Siphofaneni (S), (middleveld) and Big Bend (BB), (lowveld), (Fig 1).

### Researcher

The female researcher/focus group facilitator had lived in Eswatini for 26 years, throughout which she was involved in relocating ‘problem’ snakes and educating people about snake behaviour and characteristics.

### Data collection

Focus group discussions were conducted in each of the four rural communities and lasted between 90 and 120 minutes. They were held on Saturdays or public holidays to allow farm-workers the opportunity to attend thus addressing a limitation of a previous study [14]. Focus groups can generate rich data, providing information about attitudes, meaning and experiences in a more natural setting and allows participants to justify their positions whilst interacting. Refreshments were available from the participants’ arrival, allowing them some time to relax first and become acquainted. The venue was a community town hall or meeting place in a tranquil setting, and chairs were arranged in a circular fashion to equally include both participants and the research team. The researcher thanked everyone for coming, introduced the project aims and the topic and the conversation was audio-recorded using the colours for speaker identification. Recordings were made on two ‘iPhone’ mobile phones and a Phillips Dictaphone for back-up purposes.

The discussions aimed to generate information about beliefs and traditions associated with snakes and snakebite together with thoughts about traditional healer and allopathic treatment in snakebite cases. The study adopted a social constructionist epistemological orientation with the aim of identifying themes concerning these aims both within and between the different focus groups.

During the discussion the interpreter immediately translated any local siSwati comments into English to facilitate the flow of conversation. The data were all transcribed verbatim from the recordings by a professional transcriber and thoroughly checked for accuracy.

### Date analysis method

The transcripts were subjected to thematic analysis [55] adopting a social constructionist perspective to consider the social and historical contexts of the individual accounts in terms of cultural beliefs and traditions. The six-staged analysis firstly involved data familiarisation through re-reading the transcripts several times. The next stage involved the identification of single data items or ‘codes’ leading to the third stage which is the identification of ‘themes’ to unite these codes. The fourth stage included a review of the themes and a comparison of them to the experience of the data for confirmation, followed by a stage of ‘theme definition’ and finally the reporting of the analysis. Thematic analysis was chosen as a technique for its flexibility, enabling it to be moulded to suit project individuality whilst maintaining a freedom of expression and the recognition of abstract themes for data interpretation. The transcripts were similarly analysed to generate themes for comparison both within and between the four groups.

## Analysis and discussion

Four themes were derived from this data; ‘beliefs and traditions’, ‘logistical issues’, ‘lack of knowledge’ and ‘parallel systems’. None of the themes indicated any gender bias.

### Beliefs and traditions

Despite only 7 out of the 61 snake species in Eswatini having the potential to cause severe envenomations [9] participants generally considered all snakes to be fast, venomous and likely to bite with fatal consequences. The black mamba is consistently the most feared across all three climatic regions even though it is rare in highveld areas, indicating that beliefs are unaffected by local species variation.

*‘Some poison can kill you very fast*, *like that of the mamba*. *A mamba is the most dangerous of all the snakes*.*’* (264, BB/Purple).

*‘Me*, *I believe that all snakes are dangerous*. *Because they are enemies*, *you don’t know what they are thinking*.*’* (604, M/Clear).

Snakes known as ‘ínyoka yemadloti’ are commonly believed to represent spiritual ancestors. They have a calm disposition, often appear ‘magically’, and their colour varies depending on the family clan or surname.

*‘Do you know an ancestor’s snake*?*’* (641, PP/Red). *That snake won’t bite you … it means it belong in the family*, *in the homestead*, *part of the family*,*’* (648, PP/Red). ‘*You find it coiled in the corner there*, *inside the house and it can stay there for some days without harming anyone*. *And you won’t even notice when it has gone out*.*’* (656, PP/Red). *‘There’s no harm*. *And sometimes it carries a significant colour*. *For us*, *my sister*, *they are always green*.*’* (660, PP/Red). *‘Other clans can be brown*.*’* (663, PP/Red).

Snakes are regularly used in the practice of witchcraft [16,35], this is much feared in rural and urban communities by both the rich and the poor. ‘Witchcraft’ snakes are often only seen by the person they are destined for and once they bite they disappear leaving no trace and no bite marks. Witchdoctors may also ‘create’ imaginary snakes to protect crops and fruit trees against theft. The mystery and apprehension surrounding snakes are important tools for witchcraft, the practice is illegal in Eswatini, so ‘witching’ is a secret but lucrative business since ‘spells’ are expensive [56]. Whilst the role of most healers is to treat illness and counteract witchcraft where necessary, some are tempted into its practice. Many healers consider this an abuse and misuse of traditional methods.

*‘… There are these traditional healers and these traditional healers don’t usually go for the training*. *They take their training from their forefathers*. *But there is something about them*. *They take from the witches and from the doctors and they witch*. *They are the witches*. *The witches don’t heal*. *They are separate*. *They just don’t heal*, *they witch*.*’* (1867, PP/Purple).

*‘… witchdoctors can take a small thread like this one*, *and apply medicine on it and that thread can turn into a snake*. *They then place the thread where they know you are going to walk*. *The thread turns into a snake and it will bite you to death*.*’* (1310, BB/Purple).

*‘Once bitten by this snake*, *the victim has to consult a traditional healer*.’ (1368, BB/Brown).

*‘When you go to a traditional healer*, *he is able to deal with the evil*.*’* (1022, PP/Purple). *‘… I think it was an evil snake because he saw the fruit in the tree*, *so once he was just going to get that fruit there and that fruit changed by that time*. *It’s changed by a mamba*.*’* (1027, PP/Purple). ‘*… And that snake it biting the lady and that lady was died*. *Died on the spot*.*’* (1033, PP/1033). *‘He had bewitched his tree to protect the fruit from theft*.*’* (1043, PP/Orange). *‘Magic protection*.*’* (1052, PP/Red).

The fear elicited by snakes may be linked to an evolutionary response to aid survival [18] and can result in symptoms of stress ranging from mild anxiety to severe post-traumatic stress disorder [6]. Whilst the calm, counselling nature of traditional consultations may help reduce stress symptoms, serious envenomations require antivenom to be given as a priority. Time delays incurred from travelling to healers and administering treatment may be critical, as well as the consultation time itself where bones and prayer are often used for ancestral guidance.

*‘When I pray*, *my intention is to find out the problem that the patient has*.*’* (1447, BB/Brown). *‘I pray for the patient*, *then something will tell me what medication to give to the patient*.*’* (1454, BB/Brown).

*‘When I see that its witchcraft*, *I give them medication that will remove the foreign substance from their system*.*’* (1403, BB/Brown).

Traditional treatment complications can also seriously compound the envenomation effects. Healthcare centres often refuse to help victims who admit to having used traditional remedies in case they are later blamed for a poor outcome. Traditional beliefs can be so strong that victims will therefore lie to avoid being refused care and one participant even visited the healer for scarification treatment after leaving the clinic to avoid being turned away.

*‘… I know that in most cases they will go for traditional healers*, *about 60% of them*. (43, S/Red). *Í think it’s just the belief …*’ (47, S/Red).

*‘When bitten by a snake I go to the traditional healer first before going to hospital’*. (75, BB/Clear).

Traditional snakebite treatments are varied and may differ between communities, but participants in all four communities said they would apply a tourniquet to the limb above the bite site. Usually made from string or wire these are painful and may exacerbate local symptoms [57], even leading to limb amputation. Scarification or incisions are often made around the bite with a razor blade or sharp stone to release the ‘poison’ as the wound bleeds, but these can lead to infection and multiple use of the implement increases the risk of HIV transmission. Medicines may be oral or topical, such as cremated and ground internal organs, head and venom glands of snakes. Known locally as ‘emahlungu’ it is sometimes called ‘ísibiba’ as in neighbouring South Africa. There was some confusion over whether isibiba is made from snakes or is just powdered aloe but the fact it was of ‘traditional’ origin was more important. Emahlungu and isibiba are both widely purchased from healers and used routinely for bite prevention as well as a precautionary first aid measure. Other products may cause poisoning [58], the unripe monkey orange known as ‘lihlala’ in Pigg’s Peak and ‘umkhwakhwa’ in Big Bend induces vomiting to remove the snakebite ‘poison’ [9,43]. Traditional treatment for mamba bites involves eating mud, (apparently before the mamba eats it), to slow the venom’s effect. The victim can urinate on the ground to make mud which is then mixed with aloe and eaten, however this leads to the risk of parasitic infections. Cobra venom in the eyes is often washed with the liquid from sour milk (emasi) or breast milk, but the latter may pose an HIV risk as research indicates infected serum may transmit the virus through the cornea [59].

### Logistical issues

The study found other reasons for using traditional medicine for snakebite treatment include distance to healthcare facilities, transport, administration procedures and poor antivenom access and availability.

The distance to allopathic facilities is influential in treatment choice, the closest hospital equipped for serious envenomations may be many kilometres away.

*‘… it’s because sometimes no cars for taking the person to the clinic*. *Many clinics are very far*, *then they go to the traditional healers to go and take first aids*, *then they go to the hospital sometimes*.*’* (53, S/Orange).

*‘No*, *we go to healers because they are closer than the clinic*.*’* (124, BB/Yellow).

The lack of available transport can also result in delays affecting treatment efficacy and may further increase stress. Participants reported having to first locate and then borrow or hire a vehicle which can be expensive, costing up to a month’s salary. Buses, taxis and ambulances are generally infrequent and can be unreliable.

*‘For instance*, *in this area you can wait for 8 hours for public transport and at night there is absolutely no public transport*.*’* (350, PP/Purple)

*‘… transport delays … we are putting someone on the wrong side waiting for the ambulance*. *At time you can wait eight hours*.*’* (358, PP/Red).

*‘Sunday’s there’s not a taxi*.*’* (198, M/Green).

Local clinics do not have doctors, they may be closed at night and hospitals may experience administration delays so people felt that traditional healers are generally more plentiful and accessible.

*‘The clinic doesn’t provide any help if you come at night with a snakebite*.*’* (424, Red).

*‘Yes the clinic is not very far*. *But if it’s after midnight maybe there is no car*, *the clinic is closed*, *then they must go … sometimes they go to the traditional healer while they are still looking for the car*. *Then they take the car to the hospital because the clinic is closed*.*’* (62, S/Orange).

Antivenom shortages and/or supply issues may hinder allopathic treatment and contribute to a distrust of healthcare centres. However some did feel that health centres were more reliable since first aid was almost guaranteed immediately on arrival, whereas the healer may have no snakebite medicine or may be out collecting supplies. Many also felt snake identification is important for treatment and that this was more likely to happen at allopathic centres, together with a high standard of cleanliness and hygiene.

*‘In addition*, *the hospital is stationary*, *doesn’t move yet you might not find the traditional healer sometimes*.*’* (1182, PP/Yellow).

*‘It’s better to go to the hospital because they ask you from the onset*, *the type of snake that bit you*. *They even have pictures thus they can give you the most appropriate treatment for that snakebite*.*’* (1170, PP/Green).

*‘Besides*, *they don’t protect themselves*. *So*, *it becomes hard to say*, *please wear these gloves*.*’* (477, PP/Green). *‘So it’s painful if you know that*, *eish*, *it’s not safe*. *So*, *we can never use healers in that way*.*’* (481, PP/Green).

Treatment and consultation costs contribute to the medicine choice. In the past healers did not charge and whilst consultations are occasionally still free, some participants felt they have become expensive. Reasons for this included the outsourcing of supplies rather than collecting them themselves and a lack of any regulatory body for traditional healers.

*‘I would take the first aid first*. *And the problem why I won’t go to a traditional healer is those people are very expensive*.*’* (1428, PP/Red).

*‘You just get the treatment*, *then when they’re done*, *they tell you how to say thank you*. *They don’t say the price*.*’* (269, M/Orange).

*‘It depends on where you stay*, *whether your place is close or far from the clinic*. *Secondly*, *it also depends on available funds*. *If you have the money you can go*, *but you cannot just up and go to the clinic without money to pay for the treatment*.*’* (331, BB/Blue).

*‘… do they charge the same for the witching thing as they do for healing*?*’* (1066, PP/R), *‘No*, *witches are expensive*.*’* (1068, PP/Purple), *‘Because it’s a secret thing*.*’* (1071, PP/Red), *‘… The preparations*, *it has something that takes some time to prepare*, *that’s how it gets expensive*.*’* (1079, PP/Red).

Most participants said they would go to a clinic or hospital after traditional treatment. Many felt communities would benefit from an improvement in the transport and health facility situation, making allopathic care more accessible. A counselling session with the healer and/or ‘placebo’ medication could be useful in reducing stress and anxiety whilst transport is being arranged.

### Lack of knowledge

Most participants wanted to learn more about snake identification, behaviour and snakebite. They stated that the snake is often taken to the hospital to be identified, but this could be dangerous if the snake escaped or if it was still alive. Some snakes feign death as a defence and severed heads are still able to bite [60]. A photograph could instead be sent to a local snake expert through social media and a knowledge of factors influencing snake behaviour such as diet, temperature and defence could reduce bite occurrence [12]. Healthcare workers and traditional healers were among the participants and asked many questions during the discussions. The latter were particularly interested in learning about antivenom and in other ‘western’ ways of treatment and prevention. The social workers present admitted they receive little or no snakebite training, yet were often consulted as first responders. One said she used to believe in scarification but her training had made her aware of the risks and also of the importance of using gloves.

*‘We don’t have enough education*.*’* (1533, PP/Red).

*‘If I were to be the person responsible for educations*, *I would suggest that the radio station could give [doctors] a slot*. *That there are doctors in the radio slot*.*’* (1537, PP/Red).

*‘Is there anything to keep at our homes*, *like tablets or medicine for just in case if you bite a snake*, *you take the tablets …’* (855, S/Red).

*‘Is there anything you can do to prevent snake bites with the Western*?*’* (870, S/Clear).

*‘… the people*, *if they were educated*, *being told about the treatments and preventions you can do about the snakes …’* (868, M/Clear).

*‘… When the snake was revealed at the hospital the doctors and nurses ran away because it was a huge mamba*.*’* (1791, PP/Yellow).

### Parallel systems

In Eswatini the current lack of trust and cooperation between the two treatment-pathways mean people must make a choice. Healers welcomed the opportunity to collaborate with allopathic doctors but experienced some resistance from the doctors. Community doctors should perhaps encourage collaborations with local healers to influence the popularity of allopathic treatment for medical emergencies such as a venomous snakebite. Many healers realise their treatment limitations, they may opt for allopathic treatment for their own children and often refer victims to hospital after administering their remedies. Referrals could be expedited and first aid procedures refined if healers had more knowledge and if regulations existed such as documentation of their cases and treatments. A formal registration process and standardisation of their methods, treatments, dosages and record-keeping may increase the trust between the systems and limit the number of ‘fake’ healers. The current lack of records and the lack of any price control by healers means the system is open to abuse.

*‘I think that the problem of the government doctors*, *still fighting with the traditional healers*.*’* (333, PP/Red).

*‘Do you work with the clinic personnel*?*’* (1141, S/Orange). *‘I would say I do work with them because when my kids are sick*, *I send them to the clinic*. *However*, *they refused to work together with us as they are doctors and we are traditional healers*.*’* (1145, S/Black).

*‘So the rural healers need to actually come up with the solution to say they have something … let’s have a gathering that will also help the community because they are also … they become part of … they need to know whether their beliefs … whether they believe or not*, *they just need to know what happens in clinics and what happens with traditional healers*. *So it’s a people’s choice whether they want to go there or not*. *But at least they know*. (858, M/Black).

In South Africa, standardisation and the establishment of a regulatory body have improved the collaboration between the two systems. Some of the concerns surrounding traditional medicine [10,61] have been reduced and medical certificates from registered healers are now legally valid [62]. Whilst Eswatini has a ‘Traditional Healer’s Association’ this has not yet been formalised due to the mutual distrust so the two systems are still divided. Consequently traditional methods of diagnosis, treatment and teaching in Eswatini are not standardised so a medical registration method is not yet viable. Personal discussions with various healers in Eswatini indicate their willingness for such collaboration but they feel the government doctors are hesitant for fear of losing their jobs to healers. Collaboration between the two systems in South Africa has proved useful in obstetrics [63], has significantly improved the outcome of medical conditions such as HIV and TB [64] and may similarly assist future snakebite victims.

*‘I think you are coming up with a good initiative to integrate the knowledge from both the traditional and the Western*. *So*, *if we have one operational thing*, *it’s going to help the community*.*’* (546, PP/Red).

*‘Another problem is that the healer’s medicine helps but is not measured according to the patient’s need but it’s just the same dose for everyone*.*’* (1508, PP/Clear).

‘*They refused*. *We can work with them if they want*, *however*, *when we had a meeting with them*, *together with [a healer] and other traditional healers*, *the hospitals refused to work with us*. *The problem is we cut the skin*, *yet they do not want that*. *If you arrive at the hospital with some scarification*, *they refuse to treat you*.*’* (1062, S/Black).

*‘It lies with the modern doctors who work for government*, *they do not trust traditional healers*.*’* (946, BB/Yellow).

*‘… we had our last meeting in Mbabane … they said they want nothing to do with traditional healers*, *because we are a problem and we will make them lose their jobs*.*’* (1110, S/Black).

Participants generally felt snakebite management could be more successful if the two systems were integrated. They feel the allopathic system could be improved whilst still recognising the value and importance of traditional healers and incorporating them into future strategies. The constructive engagement of healers may be beneficial for case documentation, earlier referrals of snakebite cases to hospital, and first aid education projects such as the dangers associated with performing scarification following snakebite [13,14,30,60].

Limitations of the study included the use of an interpreter during the discussions, and a translator and transcriber for the data, although the transcripts were thoroughly checked by two people. Whilst the sample size was somewhat small due to time and logistical constraints it was representative for the information being gathered. Future studies could employ participatory appraisal methods rather than using focus groups, thus involving more participants whilst allowing their free expression of ideas. The discussions could have been subjected to social desirability influences as some participants were healers or healthcare workers, and in some cases allopathic medicine may have been considered to be more acceptable to the European researcher. Future studies could expand on the results from this project and consider the impact of social stigma and age differences within communities as well as urban and rural variations. All four communities were accessible by road so had some degree of exposure to modern society, it is likely the influence of traditional beliefs would be even stronger in more remote areas.

In summary, this study investigates the issue of effective and timeous snakebite treatment within a culturally sensitive environment. Distance to and the cost of reaching appropriate healthcare facilities as well as antivenom availability should be addressed in order to improve treatment success. Educational outreach as well as a standardisation of fees and treatment practice for traditional healers could be beneficial in snakebite management. Treatment protocols could be improved by facilitating a collaborative environment between traditional and allopathic doctors to aid the outcome of victims.
